# Alterations in the *mir-15a/16-1* Loci Impairs Its Processing and Augments B-1 Expansion in De Novo Mouse Model of Chronic Lymphocytic Leukemia (CLL)

**DOI:** 10.1371/journal.pone.0149331

**Published:** 2016-03-09

**Authors:** Siddha Kasar, Chingiz Underbayev, Moinuddin Hassan, Ilko Ilev, Heba Degheidy, Steven Bauer, Gerald Marti, Carol Lutz, Elizabeth Raveche, Mona Batish

**Affiliations:** 1 New Jersey Medical School, Rutgers University, Newark, New Jersey, 07103, United States of America; 2 OSEL/CDRH/FDA White Oak, Maryland, United States of America; 3 CBER/FDA White Oak, Maryland, United States of America; Queen's University Belfast, UNITED KINGDOM

## Abstract

New Zealand Black (NZB) mice, a de novo model of CLL, share multiple characteristics with CLL patients, including decreased expression of miR-15a/16-1. We previously discovered a point mutation and deletion in the 3' flanking region of mir-16-1 of NZB and a similar mutation has been found in a small number of CLL patients. However, it was unknown whether the mutation is the cause for the reduced miR-15a/16-1 expression and CLL development. Using PCR and in vitro microRNA processing assays, we found that the NZB sequence alterations in the *mir-15a/16-1* loci result in deficient processing of the precursor forms of miR-15a/16-1, in particular, we observe impaired conversion of pri-miR-15a/16-1 to pre-miR-15a/16-1. The in vitro data was further supported by derivation of congenic strains with replaced mir-15a/16-1 loci at one or both alleles: NZB congenic mice (N^miR+/-^) and DBA congenic mice (D^miR-/-^). The level of miR-15a/16-1 reflected the configuration of the mir-15a/16-1 loci with DBA congenic mice (D^miR-/-^) showing reduced miR-15a levels compared to homozygous wild-type allele, while the NZB congenic mice (N^miR+/-^) showed an increase in miR-15a levels relative to homozygous mutant allele. Similar to Monoclonal B-cell Lymphocytosis (MBL), the precursor stage of the human disease, an overall expansion of the B-1 population was observed in DBA congenic mice (D^miR-/-^) relative to wild-type (D^miR+/+^). These studies support our hypothesis that the mutations in the *mir-15a/16-1* loci are responsible for decreased expression of this regulatory microRNA leading to B-1 expansion and CLL development.

## Background

Chronic Lymphocytic Leukemia (CLL) is a heme malignancy characterized by accumulation of CD5+ B cells in peripheral lymphoid organs, bone marrow and peripheral blood due to an apoptosis defect [1]. It accounts for 30% of all leukemia in the Western World. Mouse models of CLL have been instrumental in understanding CLL biology {Reviewed in [2]}. Our lab has long been interested in studying CLL biology using the NZB mouse model which exhibits age associated spontaneous development of CD5^+^B220^dull^IgM^+^ B-1 cell malignancy {Reviewed in [3]}.

The malignant clone in CLL (human and NZB mice) is typically a B-1 cell expressing CD5^dim^CD45R/B220^low^IgM^hi^IgD^low^CD23^-^CD11b^+/-^ [1]. B-1 cells normally constitute only 5% of total B cells with the major fraction of these cells residing in the peritoneal cavity and a smaller fraction in the spleen. Recent evidence suggests that almost all cases of CLL are preceded by an asymptomatic precursor stage of monoclonal or pauci-clonal B cell lymphocytosis (<5x10^9^/l) termed MBL [4,5]. We have recently shown that NZB mice also exhibit this precursor MBL stage, further validating it as a true model for human CLL [6]. Studies have indicated that decreased expression of miR-15a/16-1 is one of the earliest abnormalities associated with the development of MBL [7]. Chr13q14 (region that encodes *miR-15a/16-1* in humans) deletion is the most common chromosomal abnormality in CLL, occurring in 50–60% of patients [8] and is a major mechanism for reduced expression of these two microRNAs. However, miR15a/16-1 expression was found to be low even in patients without 13q14 deletions and pointed towards alternate mechanisms for miR15a/16-1 repression such as epigenetic silencing and promoter inhibition [9,10]. A T → A point mutation and G deletion on the negative strand in the 3’ flanking region of *mir-16-1* was discovered NZB mice (de novo mouse model of CLL) and was associated with 50% reduction in expression of mature miR-15a/16-1 [11–13]. A similar point mutation has been described in a small subset of CLL patients as well. However, so far a definite causative link between the presence of these mutations and miR-15a/16-1 expression and CLL development has not been established. We hypothesized that the NZB mutation and deletion inhibits efficient processing leading to reduced level of these tumor suppressor microRNAs thereby facilitating B-1 cell expansion.

microRNAs are 22 nucleotide (nt) non-coding RNAs that regulate gene expression through transcriptional repression and in some cases through transcriptional activation [14]. Since the microRNA seed sequences for target recognition are degenerate, a single microRNA can regulate multiple mRNAs and hence multiple pathways leading to pathogenesis [15]. Deciphering the mechanisms responsible for altered microRNA expression is thus very critical. Canonical microRNA processing pathway begins with RNA pol II mediated transcription of the primary transcript (pri-miR) which consists of a dsRNA stem loop flanked by ssRNA flanking regions on either side [16]. The primary transcript is further cleaved into a 55-70nt precursor transcript (pre-miR) by Drosha, an RNase III enzyme complex. Precise cleavage by Drosha-DGCR8 microprocessor complex is critical for generating the correct mature microRNA transcript in the next step [17]. The Drosha-DGCR8 complex binds the pri-miR and cleaves ~11bp from the junction of the stem loop and ssRNA flanks [18,19]. Any alterations in this region are likely to impede Drosha activity. The resulting pre-miR transcript is then transported to the cytoplasm by Exportin 5, where it is cleaved by Dicer at a precise distance from the 2nt 3’ overhang generated by Drosha cleavage to give the final mature miRNA [20].

In this present manuscript we have investigated the effect of mutations in the *mir-15a/16-1* loci on the level of mature miR-15a/16-1, the development of B-1 cell expansion and CLL using *in vitro* processing assays and *in vivo* gene replacement. We have shown that the NZB mutation and deletion in the *mir-15a/16-1* loci blocks Drosha mediated cleavage of the primary transcript. In addition these studies reveal that introduction of this single locus results in an expansion of the B-1 subpopulation and the development of early stage CLL.

## Materials and Methods

### Cell lines

NZB (murine model of human CLL) derived malignant B-1 cell line LNC (obtained from lymph node) was used as an *in vitro* model of murine CLL [21]. A20 (TIB-208, American Type Culture Collection, Manassas, VA, USA), a BALB/c B lymphoma cell line obtained from a spontaneous reticulum neoplasm was used as a non-NZB (non-CLL) control cell line [22]. These cell lines were maintained in RPMI 1640 supplemented with 10% FBS, 1% Sodium pyruvate, 1% Penicillin-Streptomycin at 37°C and 5% CO_2_.

### Mice

NZB/BINJ (Stock No.000684) and DBA/2J (Stock No.000671) breeding pairs were obtained from Jackson Laboratories. F1 (NZB x DBA/2) mice were backcrossed to the DBA/2 parental strain to generate the first backcross for DBA congenics. These mice were tested for the presence of heterozygous NZB mutation/deletion (via Sanger sequencing). Similarly, the F1 mice were crossed to the NZB parental strain to generate the first backcrosss generation for NZB congenics. In order to sequence the *mir-15a/16-1* loci, mice were ear tagged at around 5–6 weeks. Tails were snipped and used for DNA extraction using the DNeasy Blood and Tissue Kit (Qiagen, Valencia, CA). The high amount of proteinase K used for tail digestion sometimes inhibited PCR and in those cases peripheral blood was used as source of DNA. *mir-15a/16-1* was amplified using proofreading Accuprime Pfx DNA polymerase (Invitrogen, Carlsbad, CA). The PCR products were then column purified, quantified and submitted to the Molecular Resource Facility (MRF), NJMS, Newark for Sanger sequencing. A minimum concentration of 10 ng/μl of purified PCR product was necessary for good quality sequencing. Backcross mice with heterozygous *mir-15a/16-1* loci were used in the subsequent backcross. Starting at backcross 6, intercrosses were performed to generate homozygous mutant *mir-15a/16-1* loci. Refer Table A in S1 file file for primer sequence.

### MicroRNA measurement

Total RNA, including miRNA, was extracted from cells using Trizol (Invitrogen, Carlsbad, CA), according to manufacturer’s instructions. microRNA specific cDNA was prepared using the TaqMan MicroRNA Reverse Transcriptase kit (Applied Biosystems, Carlsbad, CA). The qPCR reaction was run on the Applied Biosystems 7500 Real-Time PCR Systems for 40 cycles at 60°C. The standard 2^-ΔΔCT^ method was used for relative quantitation of the microRNA levels. For real time quantitation—mmu-miR-15a (Assay ID 000389) and U6 (Assay ID 001973) (housekeeping gene) was employed. Due to the low number of cells obtained after sorting, we performed 100 cell PCR in order to measure the miR-15a/16-1 levels in the sorted sub-populations. Briefly, for the microRNA measurement, 100 cells in 4.84μl 1X PBS were heat disrupted at 95°C for 10min to release RNA and immediately kept on ice. This extract was then used to prepare cDNA using the TaqMan microRNA RT kit (Applied Biosystems, Carlsbad, CA), followed by real time PCR.

### Preparation of DNA template for processing assay from artificial chromosome prep

NZB *mir-15a/16-1* (with mutation and deletion [MD]) plus 100bp upstream and downstream sequence was cloned into a pGEM4 plasmid (Referred to as pri-miR^MD^). This construct was then subjected to sequential site directed mutagenesis to correct the mutation and deletion giving rise to the wild type (pri-miR^wt^) construct. The Quick Change II Site Directed Mutagenesis kit (Agilent, Santa Clara, CA) was used for this purpose according to the manufacturer’s instructions. Efforts to correct both the mutation and deletion in one step failed. Hence, in first step the deletion was corrected using the pri-miR^Mut^ primer set and in second step the mutation was corrected using the pri-miR^wt^ primer set. Refer Table A in S1 file for primer sequence.

### *In vitro* microRNA processing assay

pri-miR^wt^ and pri-miR^MD^ were linearized with XbaI and used as template for *in vitro* transcription. Each *in vitro* transcription reaction contained 2μl 5X Trancription buffer, 1μl 5mM cap analog (GE Amersham), 1μl 10X rNTP, 1μl T7 polymerase, 0.5μl RNasin, 100μM DTT, 4.5μl ^32^P rCTP (Perkin Elmer), 1μl linearized template. All other components were from Promega. The mixture was incubated at 37°C for 1hr and then subjected to acid phenol:chloroform extraction (Invitrogen). After the last ethanol wash, the pellet was resuspended in 15μl of RNA loading dye (Ambion) and resolved on a 5% TBE-Urea PAGE gel (300V, 1hr). The labeled transcript was then visualized by autoradiography. The autoradiogram was super-imposed on the gel and the correct sized band was excised. The gel was eluted overnight in High Salt Crush-Elution Buffer (HSCB– 50mM Tris, 400mM NaCl, 0.1% SDS) and purified by phenol-chloroform extraction. 10^5^–10^6^ cpm of each of the labeled transcript was incubated with the processing buffer for 90min at 37°C [Processing buffer composition– 2μl 100mM ATP, 3ul 200μM creatine phosphate, 64mM MgCl2, 1μl RNase inhibitor, 20μl cell extract (prepared from 10x10^6^ cells lysed with RIPA buffer), made up volume to 30μl with input transcript and nuclease free water]. The products were phenol-chloroform extracted and resolved on a 12.5% TBE-Urea PAGE gel.

### Flow cytometry procedures

For surface staining, 1x10^6^ cells were washed twice with 1X staining buffer (2% FBS, 0.1% Sodium Azide in 1X PBS). Antibody cocktail was prepared in 150μl 1X staining buffer per tube (0.8μl of each antibody per tube) and cells were incubated for 20min at 4°C, washed twice with cold staining buffer and resuspended in 350μl of the same buffer (if acquired immediately) or 350μl 2% paraformaldehyde. For cell sorting, 1X sorter buffer (1X HBSS+2% FBS+25mM EDTA+10mM HEPES) was used instead of the 1X staining buffer. For identification of B-1 cells, single-cell suspensions were made from spleen and surface stained with anti-mouse IgM APC (Caltag, Invitrogen), anti-mouse CD5 PE (Caltag, Invitrogen), anti-IgD FITC and in some cases anti-CD5-APC, anti-B220-PE-Cy7 (RA36B2 clone).

For the detection of the side population (SP), modifications of previously reported protocols were employed [23]. Briefly, 2 x 10^6^ spleen cells (RBC lysed) were washed and re-suspended in 1ml pre-warmed IMDM + 2% fetal calf serum + 10mM Hepes and incubated at 37°C in the presence of Hoechst 33342 (5μg/ml) for 90 minutes. Confirmation of SP cells was accomplished by co-incubation with Verapamil (50μM), an ABC transporter inhibitor, which blocks Hoechst exclusion and eliminates SP visibility. Cells were immediately transferred on ice, centrifuged at 1200 rpm, 4°C for 7 minutes, resuspended in 1ml cold HBSS + 2% FCS + 10mM Hepes, and 2 μg/ml propidium iodide in PBS was added. For surface staining of side population, cells were stained with Sca-1 PE, B220 FITC, CD5 APC, and c-Kit PE-Cy7 after Hoechst incubation. Detection of a SP was verified by its absence in the presence of Verapamil. All antibodies were purchased from BD Biosciences, Franklin Lakes, NJ unless specified otherwise.

All analyses were performed on a LSR II or ARIA III (BD Biosciences) flow cytometer. FACS Diva software was used for acquisition. Cytometry setup and tracking beads (CST, BD) were used to initialize PMT settings. Unstained control cells as well as single stained tubes for FITC, PE, PerCP-Cy5.5, PE-Cy7, APC, APC-Cy7, and eFluor 450 were prepared and used to setup flow cytometric compensation. In some experiments, Comp Beads (BD) were used to set the compensation and were stained according to the manufacturer’s instruction. The number of events collected in these experiments varied from 500,000 for the single tube to 1 million. Flow Jo software (Tree Star, Ashland, OR) was used for data analysis and display.

### Spectroscopic procedures

Mid-infrared spectra were collected from spleen cells from DBA mice (normal constitutive elevated levels of miR-15a/16-1) and from NZB mice (constitutive low levels of miR-15a/16-1) in transmission mode using fiber optic based Fourier transform infrared (FTIR) spectrometer (Vertex 70, Bruker Optiks, Ettlingen, Germany) [24–26]. 2μl of spleen cells in 1X PBS solution were placed on CaF2 plate and allow to dry for ~30 min under a covered area to decrease the dust landing on the plates. Each spectrum was averaged over 256 scans in the range of 800–5000 cm^-1^ at a 4 cm^-1^ resolution. Before each measurement, background spectrum of the clean surface was collected prior to sample deposition. Three trials were recorded at different positions on the sample and averaged. To remove the contribution of PBS, the PBS was dried and spectrum was subtracted from the cell spectra before analysis. Data were analyzed using OPUS 7 data collection software.

### Computer predicted structure of pre-miR-16-1

Wild type and NZB *mir-15a/16-1* sequence was analyzed using the RNA mFold software (http://mfold.rna.albany.du/?=mfold/RNA-Folding-Form) [27]. Default RNA folding parameters were employed.

### Statistics

All experiments were performed at least in triplicates to obtain standard deviations and to calculate the SEM. Two tailed Student’s *t* test or ANOVA was used wherever appropriate to determine statistical significance, *p ≤ 0*.*05*.

## Results

### Presence of mutation in *DLEU2* loci of NZB

**Fig 1:**
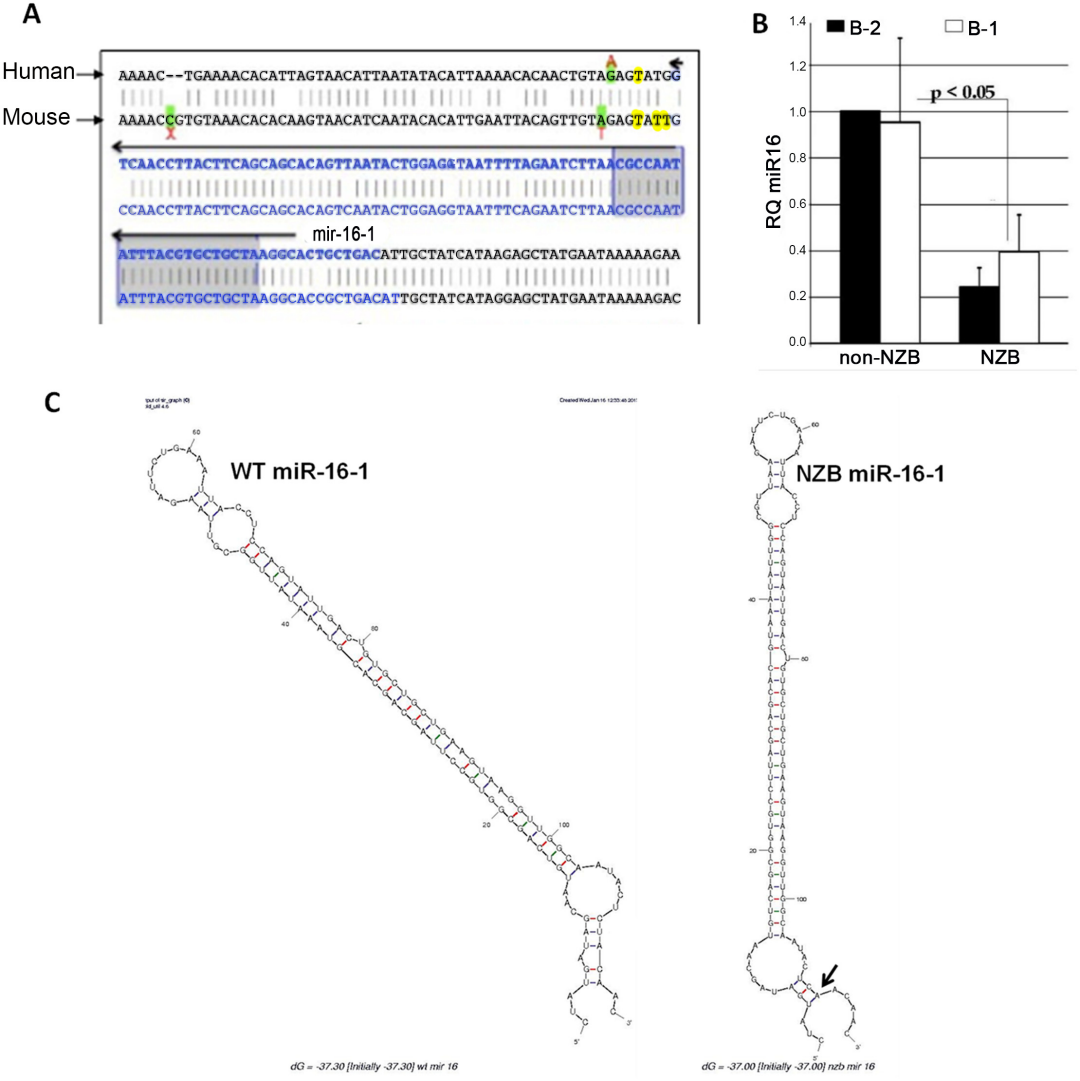
Presence of mutations in *mir-15a/16-1* loci. **A**) Partial DNA sequence of mouse and human *mir-15a/16-1* loci which is located in the intronic region of *Dleu2* in both human and mice. The mutations present in the CLL patient and the NZB mouse model of CLL are indicated. **B**) The level of mature miR-16 in DBA (non-NZB) versus NZB (CLL) spleen cells in sorted B-1 (IgM+CD5dull, B220dull) and B-2 (IgM+, CD5-, B220+) subpopulations. **C**) Predicted pre-miR-16-1 structure using the stem loop ± 11 nt sequence of wild type (left) and NZB (right) sequence using RNA mFold software. Black arrows indicate the position of the mutated base.

The sequence of NZB mice differ from all other strains tested to date and possess a mutation in the 3’flanking region of the *mir-15a/16-1* loci, similar to that reported for human CLL patients [13] (Fig 1A). In addition, NZB mice have a significant decrease in the levels of mature miR-15a and miR-16 (Fig 1B). This decrease in miR-15a/16-1 levels is not restricted to only the putative CLL precursor subpopulation of B cells (B-1) but is present in all B cells. Based on modeling analysis, there is a significant potential structural alteration in pre-miR-16-1 due to the mutation present in NZB mice (Fig 1C). While there was no difference in the free energy between the wild-type and the mutated modeled structures, the presence of the mutation alters the structure of the pre-miR-16-1 perhaps reducing accessibility to Drosha and decreasing the processivity of this microRNA precursor.

### Level of pri, pre and mature miR-15a/16-1 in NZB versus non-NZB cell line

**Fig 2:**
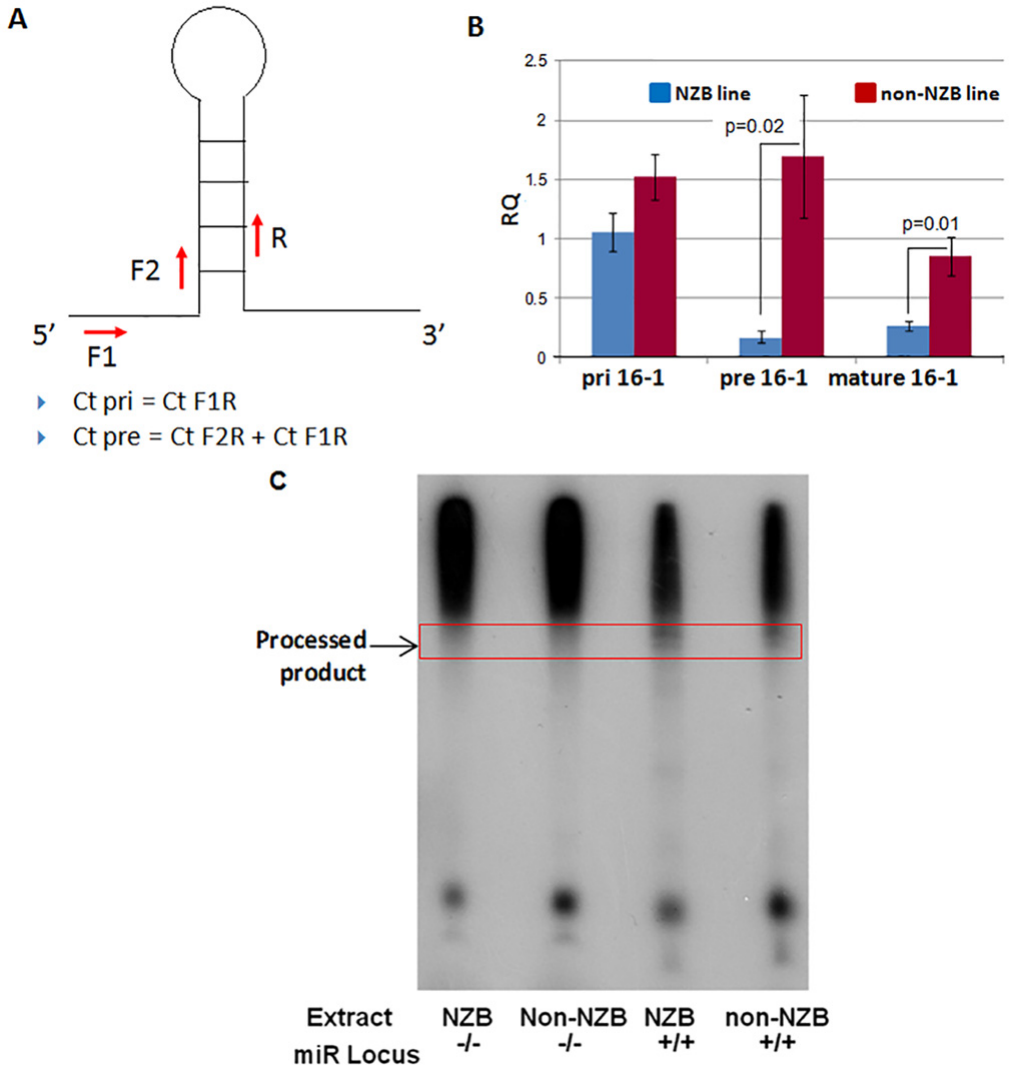
Decreased pre-miR16-1 and Mature miR16-1 in NZB. **A**) Schematic representation of the SYBR green assay for real-time measurement of primary and precursor microRNA transcripts. Employing the F1 forward and R reverse primers will prime only primary transcript, while employing F2 forward and R reverse primes both primary and precursor transcript. Hence, Ct primary = Ct F1R and Ct precursor = Ct F1R + Ct F2R. **B**) RQ values for pri-miR-16-1, pre-miR-16-1 and mature miR-16-1 in NZB (light) and non-NZB (dark) cell lines. Error bars indicate ± SEM, p values calculated using unpaired Student’s t test. **C**) The *mir-15a/16-1* loci plus 100bp upstream and downstream region containing the NZB locus (-/-) or the wild type non-NZB sequence (+/+) was cloned into a pGEM4 plasmid and used for *in vitro* processing assay. Autoradiograph of the *in vitro* processing products resolved on a 12.5% polyacrylamide gel is shown. The source of microRNA processing machinery and the genotype of the templates are indicated at the bottom.

Experiments were designed to determine the potential role of the mutation in the biogenesis of miR-15a/16-1. In order to investigate the step at which the biogenesis of miR-15a/16-1 is impaired in NZB mice, the amount of pri, pre and mature miR-16-1 in a NZB derived cell line (homozygous mutant mir-/-) and a non-NZB cell line (wild-type mir+/+) was determined. The strategy employed was based on an earlier report [28] and employed a SYBR green-PCR based strategy for the simultaneous measurement of pri and pre miR-16-1. A set of primers was designed to be specific to the double stranded portion of the stem loop (F2 and R) (Fig 2A). This set will amplify both the pri and pre transcripts. The 5’ primer (F1) of the other set binds to a region outside the pre transcript and the 3’ primer (R) binds to the stem loop, making them specific for the pri transcript only. The contribution of pri transcript to the measurement of pre transcript is then removed to calculate the actual amount of pre-miR. Since Ct value is inversely proportional to the amount of transcript, the actual Ct value for pre-miR is calculated by adding the two Ct values [29]. Only DNase (Turbo DNA-free kit, Ambion) treated RNA samples were used in these assays to prevent the amplification from genomic DNA.

The amount of pri and pre miR-16-1 were measured using the strategy outlined above, whereas the amount of mature miR-16-1 was measured using the commercially available TaqMan Assay. The expression of the primary transcript of intronic microRNA depends on the host gene transcription and splicing. *mir-15a/16-1* is encoded within the intronic region of *Dleu2*. Mutations in the vicinity of intronic microRNAs have not been shown to interfere with host gene transcription; however, they can influence splicing giving rise to aberrant levels of primary microRNA transcript [30]. No significant reduction was observed in the level of pri-miR-16-1 in the NZB B lymphoid cell line as compared to the non-NZB B lymphoid cell line. However, the amount of pre-miR-16-1 and mature miR16-1 was significantly reduced in the NZB cell line (Fig 2B). This reduction cannot be attributed to reduced stability of the pre-miR since the mutation and deletion is not present in the stem loop structure. Additionally no difference in the free energy value of pre-miR-16-1 was observed with and without the mutation using the RNA mfold program (Fig 1C).

### *In vitro* processing of *mir-15a/16-1*

Labeled microRNA primary transcripts can be processed *in vitro* using either cell extract or immunoprecipitated Drosha complex [31]. This approach enabled isolation of the effect of a particular sequence on the processing of that transcript independent of other factors. Primary miR-15a/16-1 transcripts with the NZB mutation and deletion (miR-/-) or without the mutation and deletion (miR+/+) were processed *in vitro* using either NZB or non-NZB cell extract. The miR+/+ primary transcript was cleaved much more efficiently to give the precursor transcripts than the miR-/- transcript (Fig 2C). The processing efficiency was reduced both when the source of microRNA processing machinery was NZB as well as when it was non-NZB. This indicates that the impaired processing is a function of the sequence alone but not other secondary factors.

### Spectroscopic analysis of spleen cells

**Fig 3:**
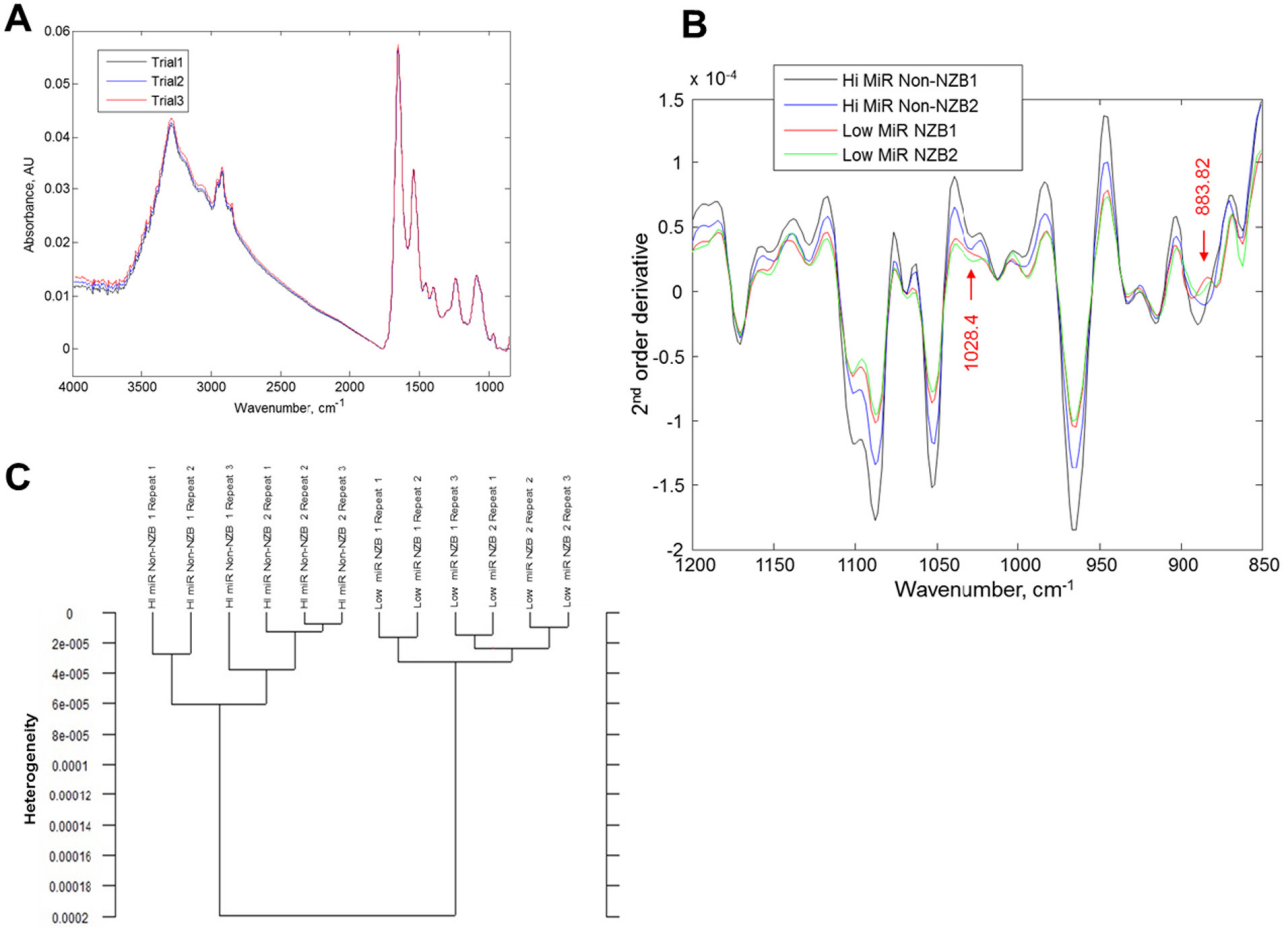
Spectroscopic Analysis (FTIR) identifies miR-15a/16-1 differences. **A**) FTIR absorption spectra of spleen cells (NZB, repeated three times). Cells were air-dried on an area of 4mm^2^ on CaF_2_ coated surface. **B**) 2^nd^ order derivative spectra of spleen cells in 1200-850cm^-1^ range. Spectral variations were observed between the non-NZB (DBA strain no CLL) with high miR-15a/16-1 levels when compared to the NZB spleen source. These were observed at 883.82 and 1028.4 cm^-1^ indicated by arrows. **C**) Hierarchical clustering was performed to investigate the effect of microRNA miR-15a/16-1 levels in spleen cells from DBA (high miR-15a/16-1) versus NZB (low miR-15a/16-1) using Ward’s algorithm and 2^nd^ order derivative spectra at 883.82 cm^-1^ and 1028cm^-1^.

We employed FTIR absorption spectra analysis to determine whether the NZB *mir-15a/16-1* mutation/deletion is associated with global macromolecular alterations. Initially, we performed whole IR spectra analysis of spleen cells to determine if the air-dried samples gave spectra which was reproducible and similar to previously reported spectra for mouse spleen (Fig 3A) [32,33]. The range associated with nucleic acids (1200–850 cm^-1^) was further analyzed and the 2^nd^ order derivative determined (Fig 3B). Following this analysis, there were two areas (1028.4 cm^-1^ and 883.8 cm^-1^) in which there was a detectable shift in the spectra between samples with high and low miR-15a/16-1. Samples expressing low miR-15a/16-1 exhibit a dip in absorbance at 1028.4 cm^-1^ indicating a reduction in nucleic acid content (Fig 3B). Cluster analysis was used to differentiate the spleen cells with High and Low microRNA based on difference of second order derivative absorption spectral data. The analysis was performed for both groups at 1028cm^-1^and 883.8 cm^-1^. Higher heterogeneity in cluster analysis demonstrates higher differences among analyzed groups. The dendrogram showed that all samples obtained from spleen cells with High and Low microRNA were successfully distinguished (Fig 3C).

### Generation of congenic mice

**Fig 4:**
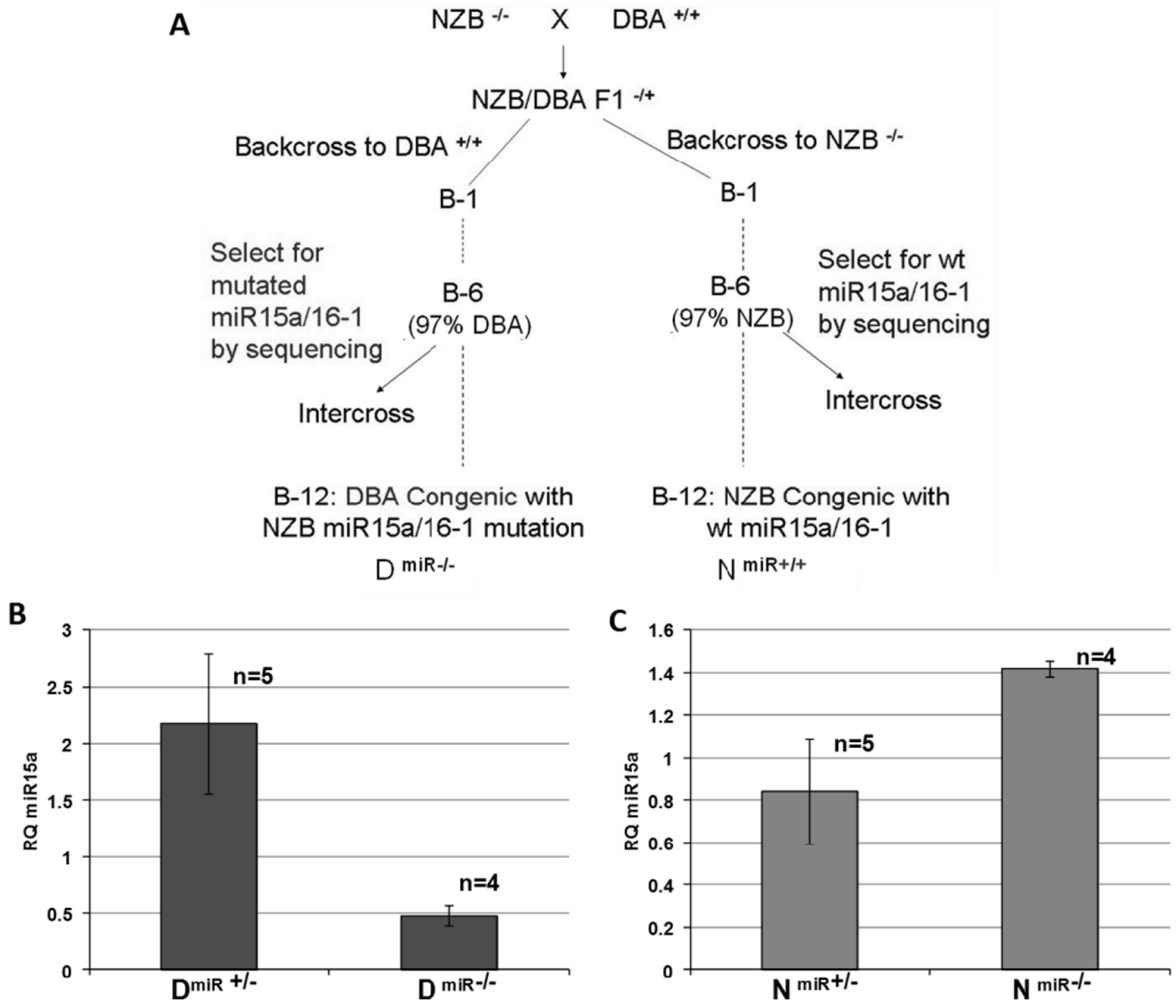
Generation of Congenic Mice. **A**) Breeding Scheme in which the two inbred parental strains NZB (which is homozygous mutant *mir-15a/16-1*) and DBA/2 (which is homozygous wild type for *mir-15a/16-1*) are crossed. Two separate congenics are established by backcrossing F1 mice to either NZB or DBA parental strain. At each backcross, progeny are selected for one copy of the each parental *mir-15a/16-1* sequence. At subsequent backcrosses mice are intercrossed and tested for the expression of either mir +/+ (DBA type), mir+/- or mir -/- (NZB type) by tail snip DNA analysis. **B**) Amount of miR-15a in DBA congenic mice which are heterozygous (D^miR+/-^) or homozygous (D^miR-/-^) for the NZB point mutation and deletion. C) Amount of miR-15a in NZB congenic mice which are heterozygous for the DBA wild-type sequence (N^miR+/-^) or remain homozygous for the NZB mutated *mir-15a/16-1* loci (N^miR-/-^) (bottom bar graph). Bar graphs indicate the amount of mature miR-15a in the peripheral blood of these mice. Level of miR-15a is expressed as a RQ value. *p<0.05, Unpaired Student’s t test, Error bars indicate mean ± SEM, n>3.

We employed the same technique that we have previously used to generate an IL-10 knockout on the NZB background [34]. Two congenic strains, were generated 1) DBA mice with NZB *mir-15a/16-1* loci (D^miR-/-^) and 2) NZB mice with the wild-type *mir-15a/16-1* loci (N^miR+/+^) (Fig 4A). Heterozygous DBA congenic mice (D^miR+/-^) were intercrossed at B6 generation. Based on Mendel’s Law of Segregation, the progeny of such an intercross have one of the three possible genotypes—D^miR+/+^ (homozygous wild-type), D^miR+/-^ (heterozygous) and D^miR-/-^ (NZB homozygous), all of which can be identified by nucleotide sequence analysis. Representative sequences are shown in **Fig A in** S1 file. To determine if the mutation and deletion in *mir-15a/16-1* (found in NZB strain) is responsible for the reduced level of mature miR-15a/16-1 observed in NZB mice, congenic mice were analyzed for the levels of miR-15a. Replacement of the wild type *mir-15a/16-1* loci with the NZB mutation and deletion gave rise to a significant reduction in the level of mature miR-15a/16-1 as compared to heterozygotes (Fig 4B). DBA congenic mice which remained homozygous wild-type (D^mir+/+^) had significantly higher levels of miR-15a in the blood than did similar congenics which had the mutation/deletion *mir-15a/16-1* loci (D^mir-/-^) (Fig 4B). Likewise, the NZB congenic mice which were no longer homozygous mutant but rather heterozygous (N^mir+/-^) had higher levels of miR-15a than NZB (N^mir-/-^) (Fig 4C **bottom**). This data suggests that the presence of the NZB mutation/deletion in the *mir-15a/16-1* loci gives rise to decreased expression of miR-15a.

### Role of miR-15a/16-1 levels on B-1 cells in congenic mice

**Fig 5:**
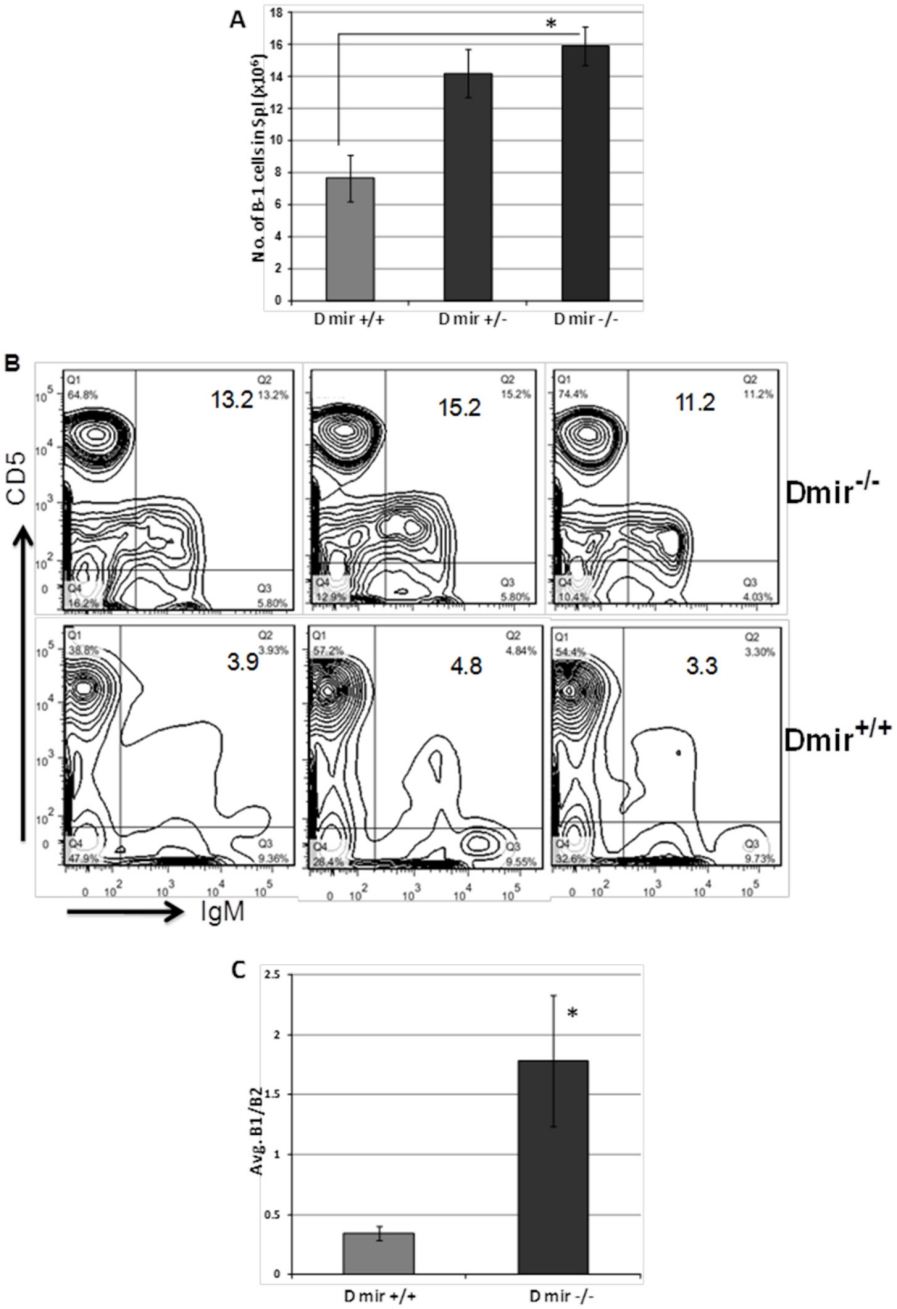
Effect of *mir-15a/16*-1 loci on B-1 expansion. **A**) Average number of total splenic B-1 cells in DBA congenic mice, *p<0.05, One-way ANOVA with Tukey’s correction. **B**) Representative flow cytometry data for the analysis of B cells in the spleens of DBA congenic mice which differ in the *mir-15a/16-1* locus. Two genotypes of DBA congenic mice were analyzed for B cell subpopulations in the lymphoid gate (B1 = CD5+, IgM+ or B2 = CD5-.IgM+) and numbers indicate the percent of B1 cells. **C**) The mean ratio of B1/B2 ± SD in congenic mice (12–18 mo of age), *p<0.05, DBA mir +/+ n = 5, DBA mir -+/- n = 3, DBA miR -/- n = 7.

CLL in both mice and humans is due to increased numbers of CD5+ B cells in blood, bone marrow, spleen and lymph node. In addition, decreased levels of miR-15a/16-1 are found in the majority of CLL. We wished to determine if decreased miR-15a/16-1 alone could result in alterations in B cells. In order to examine the effect of the *mir-15a/16-1* mutation status on B-1 expansion, cells from tissue sources were examined by flow cytometry. The percentage of B-1 cells was related to the *mir-15a/16-1* loci. DBA congenic mice possessing mutations in both alleles of the *mir-15a/16-1* loci (D^mir-/-^) had pronounced expansion of B-1 cells in the spleen (Fig 5A and 5B) relative to congenic mice with wild-type *mir-15a/16-1* (D^mir+/+^). The D^mir-/-^ congenics have the lowest levels of miR-15a (Fig 4B) and high B1/B2 ratio (Fig 5C). Thus, introduction of the NZB *mir-15a/16-1* loci to a wild-type strain is sufficient to give rise to a B-1 cell expansion.

### Effect of NZB miR-15a/16-1 locus on Side Population (SP) cells

**Fig 6:**
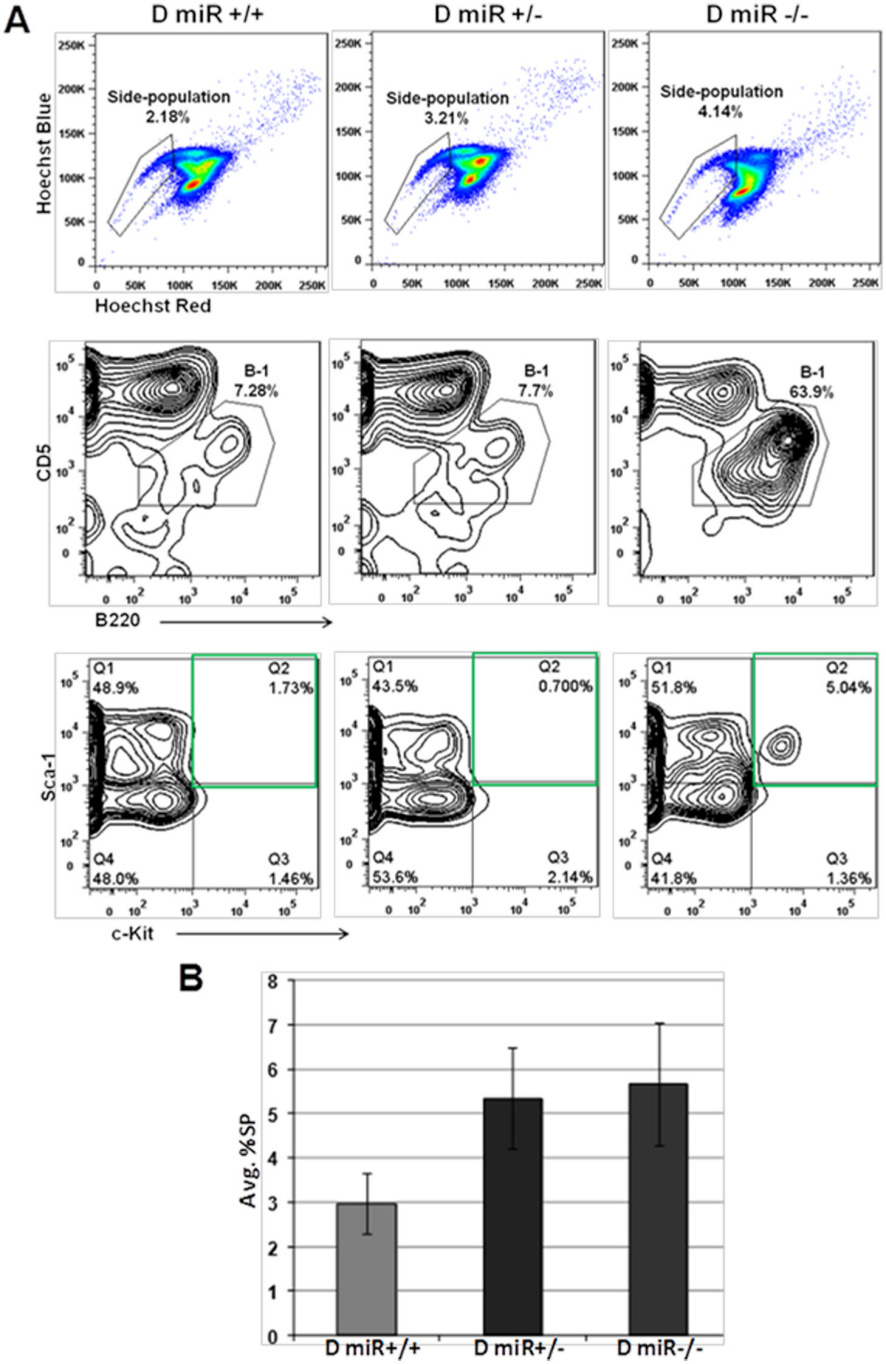
Side-population and SP B-1 in relation to *miR-15a/16-1* status. Spleen cells obtained from DBA congenic mice which differed in the *mir-15a/16-1* loci were analyzed for the presence of the sidepopulation (SP). **A) Top**—SP cells were visualized using Hoechst staining. The gates were drawn based on the blockage of dye efflux using verapamil in the corresponding mouse strain. **Middle**–The SP cells were further gated on CD5 and B220 to visualize the subpopulations in the SP fraction. Numbers indicate the percentage of CD5^+^B220^dull/+^ (B-1 cells) in D^miR+/+^ (left), D^miR+/-^ (middle) and D^miR-/-^ (right). **Bottom**—Contour plot of Sca-1 and c-Kit stained cells in the SP gate. The Sca-1^+^c-Kit^+^ population is highlighted with a green box. **B**) Average percentage of SP cells in D^miR+/+^, D^miR+/-^ and D^miR-/-^, n = 3 per group.

Since, both CLL patients and the NZB model of CLL demonstrate the expansion of a unique population of cells referred to as the side population (SP) [6,35]; this population was examined in the DBA congenics. The SP fraction contains cells that have the ability to efflux dyes, and it is this property that has been exploited for their visualization [36,37]. Spleen cells were stained with Hoechst 33342 and the SP gate was drawn by comparing with the respective (Hoechst +Verapamil tube (data not shown). The percentage of SP almost doubled in D^miR-/-^ as compared to D^miR+/+^ while the heterozygote was intermediate (Fig 6A **Top**). Further dissection of the sub-populations in the SP revealed that 64% of the SP cells in D^miR-/-^ were B-1 cells (CD5^+^B220^dull/+^) versus only 7.7% and 7.3% in heterozygous (D^miR+/-^) and wild type homologous (D^miR+/+^) miR-15a/16-1 loci respectively (Fig 6A **Middle**). The percentage of primitive Sca-1^+^c-Kit^+^ cells was also increased in the homozygous NZB *mir-15a/16-1* loci (D^miR-/-^) (Fig 6A **Bottom**). An increase in SP cells (which are enriched in stem cells) was observed in congenic mice with reduced miR-15a/16-1 levels (Fig 6B). The side-population may also contain cells that are more resistant to chemotherapy based on the increased ability to efflux. Interestingly in the cell source with reduced miR-15a/16-1 (D^miR-/-^) the percentage of B-1 cells was greatly increased.

## Discussion

Raveche et al reported the discovery of a germline point mutation and deletion (T → A and G deletion on the negative strand) in the 3’ flanking region of *miR-16-1* of NZB mice [12]. Calin et al also reported a similar point mutation (G→A on negative strand) in the 3’ flanking region of miR-16-1 in a small population of CLL patients [13]. Although these alterations have been associated with the reduced expression of mature miR-15a/16-1 [12,13], no causal relationship has been established. The presence of such conserved alterations in both mouse and human suggest that miR-15a/16-1 expression is important for CLL pathogenesis. We have recently shown the therapeutic benefit of restoring miR-15a/16-1 expression by two independent methods– 1) *In vivo* lentiviral delivery; 2) De-repression of the host gene promoter using HDAC inhibitor [10,38]. Indeed, microRNA dysregulation plays a central role in pathogenesis of a number of diseases including cancer and understanding the mechanism responsible for this dysregulation is imperative for therapeutic targeting. microRNA expression has been shown to be regulated at multiple levels. microRNA sequence alterations have been linked to blocking of either of the two processing steps (Drosha or Dicer cleavage) [39,40]. These sequence alterations occur quite commonly in cancer and have been shown to contribute to tumor development and progression [41,42]. Hence here we have studied the CLL associated mutation and deletion in *mir-15a/16-1* in relation to the effect of these mutations on microRNA processing and B-1 expansion.

The reduced mature miR-15a/16-1 expression associated with the NZB loci could be attributed to either reduced transcription or to impaired processing. The amount of pri-miR-15a/16-1 was not significantly reduced in the cells bearing the NZB *mir-15a/16-1* loci as compared to the wild type cells. Thus, the mutation and deletion does not interfere with the transcription or splicing of the host *Dleu2* gene to produce the primary transcript. However, the amount of pre-miR-15a/16-1 was significantly reduced in the NZB cells as compared to the non-NZB (wild-type in *mir-15a/16-1*) cells. We hypothesized that this mutation and deletion either inhibits Drosha mediated cleavage of the primary transcript or decreases the stability of the precursor transcript. However, the mutation and deletion is 5nt downstream of the pre-transcript and is unlikely to influence its stability.

Impaired Drosha cleavage is the most likely event responsible for the reduced pre-miR. The precise molecular mechanism of Drosha activity is still not completely understood. However, studies have shown that Drosha cleaves 11bp from the stem loop-ssRNA junction [18,43]. The NZB mutation falls within this range and could potentially inhibit Drosha mediated cleavage. Computational folding of pre-miR-16-1 with the NZB mutation gave rise to a potential altered structure when compared to the wild-type sequence predicted structure. This predicted structural change occurs right at the Drosha cleavage site. Changes in absorbance at 883 cm^-1^ observed by mid-IR spectroscopy in mutant miR-15a/16-1 cells versus the wildtype is further indicative of structural changes. Processing of the *in vitro* transcribed miR-15a/16-1 with the mutation and deletion mimics the NZB phenotype (decreased pre-miR) whereas the wild type primary transcript is cleaved to precursor form. The processing efficiency was independent of the source of the microRNA processing machinery, indicating that the phenotype is dependent on the DNA sequence alteration alone. Reduced mature miR-15a/16-1 in DBA congenic mice (D^miR-/-^) and its reverse in NZB congenic mice (N^miR+/-^) is a further proof that the NZB *mir-15a/16-1* locus is the cause for reduction in mature miR-15a/16-1. Given the synteny between mouse and human in this loci, it is likely that a similar processivity block is present in the CLL patients with germline mutations in miR-16-1 as reported by Calin et al [13,44].

Previous studies have shown that reduced miR-15a/16-1 promotes CLL cell survival and increases chemoresistance. In addition, findings from the DBA congenic mice show that downregulation of miR-15a/16-1 plays an important role in B-1 expansion. B-1 expansion was observed not only in the spleen but also in the PWC and PBMC of these mice (**Fig B in** S1 file). This was followed by an increase in the percentage of cells in the SP (side-population) fraction, an observation that is consistent with previously reported increases in SP in NZB mice with age [6]. SP is a special population of cells that has the capacity to efflux dyes and other xenobiotics [45]. SP has been shown to contain primitive cells that can function as a cancer stem cell in acute myelogenous leukemia (AML) [46]. It is interesting to note that the percentage of Sca-1+ ckit+ primitive cells and B-1 cells in SP fraction was much higher in D^miR-/-^ than the D^miR+/+^. However, whether or not the SP fraction contains the CLL cancer stem cell warrants further investigation. Nevertheless, SP cells are important for CLL biology. Their resistance to chemotherapy in CLL has been well documented [47,48]. Persistence of this drug resistant minor population has been associated with increased risk of relapse [49].

Unpublished data from Raveche Lab indicates that reduced expression of miR-15a/16-1 skews the differentiation of hematopoietic stem cells towards B-1 progenitors via aberrant expression of PU.1, a transcription factor that is critical for B lineage development [50]. Hence, the B-1 expansion observed in the congenic mice could be attributed to such skewing.

Preliminary results also shown that mid-IR spectroscopy may be used to differentiate between cells expressing high and low levels miR-15a/16-1 as seen from our hierarchical cluster analysis. Given the frequent deletions in this locus in CLL patients, this approach has the potential to be developed for a screening tool. Several groups have proposed the use of mid-IR spectroscopy as a diagnostic tool in the past {Reviewed in [51]}.

In summary, the results presented here show that the alterations found in the *mir-15a/16-1* loci of NZB lead to decreased processivity resulting in decreased expression of mature miR-15a and miR-16-1, which in turn gives rise to B-1 expansion.

## Supporting Information

S1 File Table A. List of the primers used in this study. **Fig A. Genotyping of Congenic Mice using Sanger Sequencing**: The miR-15a/16-1 locus was amplified from tail DNA and visualized using FinchTV software. The blue and green box indicates the site of mutation and deletion respectively. Samples shown from top to bottom are control DBA mouse (homozygous, wild type, DBA^miR+/+^), DBA congenic with wild type homozygous locus (D^miR+/+^), DBA congenic with heterozygous locus (D^miR+/-^), DBA congenic with homozygous mutation and deletion (D^miR-/-^), NZB congenic with heterozygous locus (N^miR+/-^) and control NZB mouse (homozygous mutation and deletion, NZB^miR-/-^). **Fig B. Alterations in B1 cell Population with Genotype**: Representative flow cytometry data for the analysis of percentage of B-1 cells in spleen (A), PWC (B) and PBMC (C). In each panel, the CD19+ population (single color histogram, top row) was gated on IgM and IgD to (contour plots, bottom row) to obtain percentage of B-1 cells in the B cell compartment. The *miR-15a/16-1* mutation status is indicated above each histogram, n = 1 per group.
(DOCX)

## References

[1] Dillman RO (2008) Immunophenotyping of Chronic Lymphoid Leukemias. Journal of Clinical Oncology 26: 1193–1194. 10.1200/JCO.2007.14.1424 18323542

[2] Simonetti G, Bertilaccio MT, Ghia P, Klein U (2014) Mouse models in the study of chronic lymphocytic leukemia pathogenesis and therapy. Blood 124: 1010–1019. 10.1182/blood-2014-05-577122 25006127

[3] Scaglione BJ, Salerno E, Balan M, Coffman F, Landgraf P, Abbasi F, et al (2007) Murine models of chronic lymphocytic leukaemia: role of microRNA-16 in the New Zealand Black mouse model. Br J Haematol 139: 645–657. 17941951 10.1111/j.1365-2141.2007.06851.x PMC2692662

[4] Caporaso NE, Marti GE, Landgren O, Azzato E, Weinberg JB, Goldin L, et al (2010) Monoclonal B cell lymphocytosis: clinical and population perspectives. Cytometry B Clin Cytom 78 Suppl 1: S115–119. 10.1002/cyto.b.20555 20839332 PMC7282703

[5] Landgren O, Albitar M, Ma W, Abbasi F, Hayes RB, Ghia P, et al (2009) B-cell clones as early markers for chronic lymphocytic leukemia. N Engl J Med 360: 659–667. 10.1056/NEJMoa0806122 19213679 PMC7015348

[6] Salerno E, Yuan Y, Scaglione BJ, Marti G, Jankovic A, Mazzella F, et al (2010) The New Zealand black mouse as a model for the development and progression of chronic lymphocytic leukemia. Cytometry B Clin Cytom 78 Suppl 1: S98–109. 10.1002/cyto.b.20544 20839343 PMC2963456

[7] Lanasa MC, Allgood SD, Volkheimer AD, Gockerman JP, Whitesides JF, Goodman BK, et al (2010) Single-cell analysis reveals oligoclonality among 'low-count' monoclonal B-cell lymphocytosis. Leukemia 24: 133–140. 10.1038/leu.2009.192 19946263 PMC2806490

[8] Dohner H, Stilgenbauer S, Benner A, Leupolt E, Krober A, Bullinger L, et al (2000) Genomic aberrations and survival in chronic lymphocytic leukemia. N Engl J Med 343: 1910–1916. 11136261 10.1056/NEJM200012283432602

[9] Sampath D, Liu C, Vasan K, Sulda M, Puduvalli VK, Wierda WG, et al (2012) Histone deacetylases mediate the silencing of miR-15a, miR-16, and miR-29b in chronic lymphocytic leukemia. Blood 119: 1162–1172. 10.1182/blood-2011-05-351510 22096249 PMC3277352

[10] Kasar S, Underbayev C, Yuan Y, Hanlon M, Aly S, Khan H, et al (2014) Therapeutic implications of activation of the host gene (Dleu2) promoter for miR-15a/16-1 in chronic lymphocytic leukemia. Oncogene 33: 3307–3315. 10.1038/onc.2013.291 23995789 PMC4508006

[11] Salerno E, Yuan Y, Scaglione BJ, Marti G, Jankovic A, Mazzella F, et al (2010) The New Zealand black mouse as a model for the development and progression of chronic lymphocytic leukemia. Cytometry Part B, Clinical cytometry 78 Suppl 1: S98–109. 10.1002/cyto.b.20544 20839343 PMC2963456

[12] Raveche ES, Salerno E, Scaglione BJ, Manohar V, Abbasi F, Lin YC, et al (2007) Abnormal microRNA-16 locus with synteny to human 13q14 linked to CLL in NZB mice. Blood 109: 5079–5086. 17351108 10.1182/blood-2007-02-071225 PMC1890829

[13] Calin GA, Ferracin M, Cimmino A, Di Leva G, Shimizu M, Wojcik SE, et al (2005) A MicroRNA signature associated with prognosis and progression in chronic lymphocytic leukemia. N Engl J Med 353: 1793–1801. 16251535 10.1056/NEJMoa050995

[14] Vasudevan S, Tong Y, Steitz JA (2007) Switching from repression to activation: microRNAs can up-regulate translation. Science 318: 1931–1934. 18048652 10.1126/science.1149460

[15] Lambert NJ, Gu SG, Zahler AM (2011) The conformation of microRNA seed regions in native microRNPs is prearranged for presentation to mRNA targets. Nucleic Acids Res 39: 4827–4835. 10.1093/nar/gkr077 21335607 PMC3113585

[16] Lee Y, Kim M, Han J, Yeom KH, Lee S, Baek SH, et al (2004) MicroRNA genes are transcribed by RNA polymerase II. EMBO J 23: 4051–4060. 15372072 10.1038/sj.emboj.7600385 PMC524334

[17] Han J, Lee Y, Yeom KH, Kim YK, Jin H, Kim VN (2004) The Drosha-DGCR8 complex in primary microRNA processing. Genes Dev 18: 3016–3027. 15574589 10.1101/gad.1262504 PMC535913

[18] Han J, Lee Y, Yeom KH, Nam JW, Heo I, Rhee JK, et al (2006) Molecular basis for the recognition of primary microRNAs by the Drosha-DGCR8 complex. Cell 125: 887–901. 16751099 10.1016/j.cell.2006.03.043

[19] Gregory RI, Yan KP, Amuthan G, Chendrimada T, Doratotaj B, Cooch N, et al (2004) The Microprocessor complex mediates the genesis of microRNAs. Nature 432: 235–240. 15531877 10.1038/nature03120

[20] Bohnsack MT, Czaplinski K, Gorlich D (2004) Exportin 5 is a RanGTP-dependent dsRNA-binding protein that mediates nuclear export of pre-miRNAs. RNA 10: 185–191. 14730017 10.1261/rna.5167604 PMC1370530

[21] Peng B, Sherr DH, Mahboudi F, Hardin J, Wu YH, Sharer L, et al (1994) A cultured malignant B-1 line serves as a model for Richter's syndrome. Journal of immunology 153: 1869–1880. 8046247

[22] Kim KJ, Kanellopoulos-Langevin C, Merwin RM, Sachs DH, Asofsky R (1979) Establishment and characterization of BALB/c lymphoma lines with B cell properties. J Immunol 122: 549–554. 310843

[23] Challen GA, Boles N, Lin KK, Goodell MA (2009) Mouse hematopoietic stem cell identification and analysis. Cytometry A 75: 14–24. 10.1002/cyto.a.20674 19023891 PMC2640229

[24] Naumann D, Helm D, Labischinski H (1991) Microbiological characterizations by FT-IR spectroscopy. Nature 351: 81–82. 1902911 10.1038/351081a0

[25] Sahu R, Mordechai S (2005) Fourier transform infrared spectroscopy in cancer detection. Future Oncol 1: 635–647. 16556041 10.2217/14796694.1.5.635

[26] Hassan M, Tan X, Welle E, Ilev I (2013) Fiber-optic Fourier transform infrared spectroscopy for remote label-free sensing of medical device surface contamination. Rev Sci Instrum 84: 053101 10.1063/1.4803182 23742526

[27] Zuker M (2003) Mfold web server for nucleic acid folding and hybridization prediction. Nucleic Acids Res 31: 3406–3415. 12824337 10.1093/nar/gkg595 PMC169194

[28] Schmittgen TD, Lee EJ, Jiang J, Sarkar A, Yang L, Elton TS, et al (2008) Real-time PCR quantification of precursor and mature microRNA. Methods 44: 31–38. 18158130 10.1016/j.ymeth.2007.09.006 PMC2663046

[29] Schmittgen TD, Livak KJ (2008) Analyzing real-time PCR data by the comparative C(T) method. Nat Protoc 3: 1101–1108. 18546601 10.1038/nprot.2008.73

[30] Janas MM, Khaled M, Schubert S, Bernstein JG, Golan D, Veguilla RA, et al (2011) Feed-forward microprocessing and splicing activities at a microRNA-containing intron. PLoS Genet 7: e1002330 10.1371/journal.pgen.1002330 22028668 PMC3197686

[31] Lee Y, Ahn C, Han J, Choi H, Kim J, Yim J, et al (2003) The nuclear RNase III Drosha initiates microRNA processing. Nature 425: 415–419. 14508493 10.1038/nature01957

[32] Gaigneaux A, Ruysschaert JM, Goormaghtigh E (2002) Infrared spectroscopy as a tool for discrimination between sensitive and multiresistant K562 cells. Eur J Biochem 269: 1968–1973. 11952799 10.1046/j.1432-1033.2002.02841.x

[33] Ozek NS, Tuna S, Erson-Bensan AE, Severcan F (2010) Characterization of microRNA-125b expression in MCF7 breast cancer cells by ATR-FTIR spectroscopy. Analyst 135: 3094–3102. 10.1039/c0an00543f 20978686

[34] Czarneski J, Lin YC, Chong S, McCarthy B, Fernandes H, Parker G, et al (2004) Studies in NZB IL-10 knockout mice of the requirement of IL-10 for progression of B-cell lymphoma. Leukemia 18: 597–606. 14712288 10.1038/sj.leu.2403244

[35] Foster AE, Okur FV, Biagi E, Lu A, Dotti G, Yvon E, et al (2010) Selective elimination of a chemoresistant side population of B-CLL cells by cytotoxic T lymphocytes in subjects receiving an autologous hCD40L/IL-2 tumor vaccine. Leukemia 24: 563–572. 10.1038/leu.2009.281 20072155 PMC2836398

[36] Goodell MA, Rosenzweig M, Kim H, Marks DF, DeMaria M, Paradis G, et al (1997) Dye efflux studies suggest that hematopoietic stem cells expressing low or undetectable levels of CD34 antigen exist in multiple species. Nat Med 3: 1337–1345. 9396603 10.1038/nm1297-1337

[37] Petriz J (2007) Flow cytometry of the side population (SP). Curr Protoc Cytom Chapter 9: Unit9 23. 10.1002/0471142956.cy0923s39 18770857

[38] Kasar S, Salerno E, Yuan Y, Underbayev C, Vollenweider D, Laurindo MF, et al (2012) Systemic in vivo lentiviral delivery of miR-15a/16 reduces malignancy in the NZB de novo mouse model of chronic lymphocytic leukemia. Genes Immun 13: 109–119. 10.1038/gene.2011.58 21881595 PMC3516396

[39] Jazdzewski K, Murray EL, Franssila K, Jarzab B, Schoenberg DR, de la Chapelle A (2008) Common SNP in pre-miR-146a decreases mature miR expression and predisposes to papillary thyroid carcinoma. Proceedings of the National Academy of Sciences 105: 7269–7274. 10.1073/pnas.0802682105 PMC2438239 18474871

[40] Kawahara Y, Zinshteyn B, Chendrimada TP, Shiekhattar R, Nishikura K (2007) RNA editing of the microRNA-151 precursor blocks cleavage by the Dicer-TRBP complex. Embo Reports 8: 763–769. 17599088 10.1038/sj.embor.7401011 PMC1978079

[41] Xing J-L, Wan S, Zhou F, Qu F, Li B, Myers RE, et al (2011) Genetic polymorphisms in pre-microRNA genes as prognostic markers of colorectal cancer. Cancer Epidemiology Biomarkers & Prevention. 10.1158/1055-9965.EPI-11-0624 PMC3253954 22028396

[42] Wu M, Jolicoeur N, Li Z, Zhang L, Fortin Y, L'Abbe D, et al (2008) Genetic variations of microRNAs in human cancer and their effects on the expression of miRNAs. Carcinogenesis 29: 1710–1716. 10.1093/carcin/bgn073 18356149

[43] Wu H, Ye C, Ramirez D, Manjunath N (2009) Alternative processing of primary microRNA transcripts by Drosha generates 5' end variation of mature microRNA. PLoS One 4: e7566 10.1371/journal.pone.0007566 19859542 PMC2762519

[44] Allegra D, Bilan V, Garding A, Dohner H, Stilgenbauer S, Kuchenbauer F, et al (2014) Defective DROSHA processing contributes to downregulation of MiR-15/-16 in chronic lymphocytic leukemia. Leukemia 28: 98–107. 10.1038/leu.2013.246 23974981

[45] Hirschmann-Jax C, Foster AE, Wulf GG, Nuchtern JG, Jax TW, Gobel U, et al (2004) A distinct “side population” of cells with high drug efflux capacity in human tumor cells. Proc Natl Acad Sci U S A 101: 14228–14233. 15381773 10.1073/pnas.0400067101 PMC521140

[46] Moshaver B, van Rhenen A, Kelder A, van der Pol M, Terwijn M, Bachas C, et al (2008) Identification of a small subpopulation of candidate leukemia-initiating cells in the side population of patients with acute myeloid leukemia. Stem Cells 26: 3059–3067. 10.1634/stemcells.2007-0861 19096043

[47] Gross E, L'Faqihi-Olive FE, Ysebaert L, Brassac M, Struski S, Kheirallah S, et al (2010) B-chronic lymphocytic leukemia chemoresistance involves innate and acquired leukemic side population cells. Leukemia 24: 1885–1892. 10.1038/leu.2010.176 20827287

[48] Hudecek M, Schmitt TM, Baskar S, Lupo-Stanghellini MT, Nishida T, Yamamoto TN, et al (2010) The B-cell tumor—associated antigen ROR1 can be targeted with T cells modified to express a ROR1-specific chimeric antigen receptor. Blood 116: 4532–4541. 10.1182/blood-2010-05-283309 20702778 PMC2996114

[49] Foster AE, Okur FV, Biagi E, Lu A, Dotti G, Yvon E, et al (2009) Selective depletion of a minor subpopulation of B-chronic lymphocytic leukemia cells is followed by a delayed but progressive loss of bulk tumor cells and disease regression. Mol Cancer 8: 106 10.1186/1476-4598-8-106 19922650 PMC2784756

[50] Underbayev C, Kasar S, Degheidy H, Marti G, Lightfoote M, Raveche E (2014) Abstract 4791: The role of microRNA-15a/16 in early B1 cell development in a mouse model of chronic lymphocytic leukemia. Cancer Res 74: 4791.

[51] Simonova D, Karamancheva I (2013) Application of Fourier Transform Infrared Spectroscopy for Tumor Diagnosis. Biotechnology & Biotechnological Equipment 27: 4200–4207.

