# Supplementary material for: Alterations in the mir-15a/16-1 Loci Impairs Its Processing and Augments B-1 Expansion in De Novo Mouse Model of Chronic Lymphocytic Leukemia (CLL)
Source: PLoS One. 2016 Mar 9;11(3):e0149331. doi: 10.1371/journal.pone.0149331 (PMC4784815; doi:10.1371/journal.pone.0149331)
Supplement: S1 File — Fig A. Genotyping of Congenic Mice using Sanger Sequencing: The miR-15a/16-1 locus was amplified from tail DNA and visualized using FinchTV software. The blue and green box indicates the site of mutation and deletion respectively. Samples shown from top to bottom are control DBA mouse (homozygous, wild type, DBAmiR+/+), DBA congenic with wild type homozygous locus (DmiR+/+), DBA congenic with heterozygous locus (DmiR+/-), DBA congenic with homozygous mutation and deletion (DmiR-/-), NZB congenic with heterozygous locus (NmiR+/-) and control NZB mouse (homozygous mutation and deletion, NZBmiR-/-). Fig B. Alterations in B1 cell Population with Genotype: Representative flow cytometry data for the analysis of percentage of B-1 cells in spleen (A), PWC (B) and PBMC (C). In each panel, the CD19+ population (single color histogram, top row) was gated on IgM and IgD to (contour plots, bottom row) to obtain percentage of B-1 cells in the B cell compartment. The miR-15a/16-1 mutation status is indicated above each histogram, n = 1 per group. (DOCX) [file pone.0149331.s001.docx]

**Table A: List of primers**

| **No.** | **Primer set** | **Forward** | **Reverse** |
| --- | --- | --- | --- |
| 1 | *miR-15a/16-1* | cctggtatgcagtggtaaggc | ctattgaggtgctaggag |
| 2 | pri-miR^Mut^ | ATGTTACTTGTGTGTTTACACGGTTTTTCTAATTGCATTAGTG | CAATACACATTGAATTACAGTTGTAGACTATTGCCAACCTTACTTC |
| 3 | pri-miR^wt^ | GGTTGGCAATACTCTACAACTGTAATTC | AACCTTACTTCAGCAGCACAGTC |
| 4 | mmu-pri-miR15a | CAGCACTGTATTAAGTTTTCTGTACGC | GCAGCACAGTATGGCCTG |
| 5 | mmu-pre-miR-15a | GTAGCAGCACATAATGGTTTGTG | GCAGCACAGTATGGCCTG |
| 6 | mmu-pri-miR-16-1 | GCAGCACGTAAATATTGGCGT | CAGACACAATATGTAGAGCGTTG |
| 7 | mmu-pre-miR-16-1 | GCAGCACGTAAATATTGGCGT | CAGCAGCACAGTCAATACTGGAG |

**Fig A. Genotyping of Congenic Mice using Sanger Sequencing**

**Fig B. Alteration in B1 cell Population with Genotype**
